# Associations of outdoor activity and screen time with adiposity: findings from rural Chinese adolescents with relatively low adiposity risks

**DOI:** 10.1186/s12889-020-09897-7

**Published:** 2020-11-23

**Authors:** Youjie Zhang, Xiaofan Zhang, Jun Li, Hua Zhong, Chen-Wei Pan

**Affiliations:** 1 School of Public Health, Medical College of Soochow University, 199 Ren Ai Road, Suzhou, 215123 China; 2 Department of Ophthalmology, the Second People’s Hospital of Yunnan Province, Kunming, 650021 China; 3 Department of Ophthalmology, the First Affiliated Hospital of Kunming Medical University, Kunming, 650032 China

**Keywords:** Early adolescents, Rural, Overweight and obesity, Waist circumference, Outdoor activity, Screen time

## Abstract

**Background:**

Whether and to what extent outdoor activity and screen time are relevant to adiposity among rural adolescents remain largely unknown as most of relevant evidence was generated from high-income countries and urban areas. This study aimed to investigate associations of outdoor activity and screen time with adiposity among early adolescents living in rural southwest China.

**Methods:**

In this cross-sectional study, seventh graders (*n* = 2264) were recruited from 10 middle schools of a rural county. Overweight and obesity was assessed using adolescents’ body mass index and waist circumference. Adolescents’ outdoor activity and screen time were measured using self-reported daily time spending on outdoor activity, watching TV, playing video games, and computers on weekdays and weekends, respectively.

**Results:**

The prevalence of overweight/obesity and high waist circumferences were 8.0 and 4.9% and were higher among those from one-child families and with parents having high school or higher education and whose fathers were not farmers. Adolescents who did not have ≥1 h outdoor activity on weekdays were more likely to be overweight/obese (OR: 1.86, 95% CI: 1.30, 2.66) and have high waist circumferences (OR: 2.22, 95%CI: 1.39, 3.57). Adolescents who had > 2 h screen time on weekends were more likely to have high waist circumferences (OR: 2.08, 95% CI: 1.14, 3.80). Lack of outdoor activity and excessive screen time also showed synergistic effects on overweight/obesity (OR: 1.93. 95% CI: 1.15, 3.24) and high waist circumferences (OR: 3.02, 95% CI: 1.54, 5.94).

**Conclusions:**

Lack of outdoor activity and excessive screen time were relevant to adiposity among rural Chinese adolescents even when the obesity prevalence was low. Efforts to promote active lifestyles may help prevent rural adolescents from losing their advantage in the era of the global obesity epidemic.

## Background

Globally, childhood obesity affected about 124 million children and adolescents in 2016, and more than half of which were from low- and middle-income countries [1]. Different from high-income countries, the prevalence of childhood obesity has been higher in urban areas than rural areas in low- and middle-income countries [2]. However, this urban-rural gap is closing as childhood obesity has been growing faster in rural areas than in urban areas. For example, in China, the estimated annual increases of childhood obesity between 2005 and 2010 were 9 and 4% among rural boys and girls as compared to 3 and 2% of urban boys and girls [3]. In 2015, the urban-rural gap was no longer statistically significant at the national level [4]. This alarms the disappearing advantage in rural areas and calls for urgent efforts on preventing unhealthy weight gain among rural pediatric populations.

From an energy balance point of view, unhealthy weight gain results from long-term dynamics of positive balance between energy intake and expenditure [5, 6]. Lifestyle behaviors that related to energy balance are modifiable risk factors of obesity. Physical inactivity and excessive screen time are two major aspects of sedentary lifestyles, which are associated with adiposity along with other detrimental health implications among the young [7]. Internationally, authoritative agencies and institutions have developed guidelines that recommend at least one hour of physical activity and no more than two hours of recreational screen time for school-aged children and adolescents [8, 9]. These guidelines have been widely applied for assessing and managing sedentary behaviors. However, most of available evidence regarding childhood obesity and its relationships with sedentary behaviors came from high-income countries or relatively developed urban areas of low- and middle-income countries.

Outdoor activity is the most common form of physical activity engaged and promoted among Chinese youth [10]. The government have initiated a “National Sunshine Sports for Million Students” program since 2007 [11]. Rural adolescents have limited access to indoor sports facilities and are more likely to be physically active outdoor [12]. A national survey of Chinese children aged 6–17 years (*n* = 62, 517) found that adolescents from rural areas spent more time in outdoor activities than their counterparts living in urban areas [13]. However, the China 2018 Report Card on physical activity for children and youth indicated that only 13.1% students reported being physically active at 60 min daily, whereas 92.9% students reported having 2 h of screen time or more per day [14]. Despite of the concerning situation, whether and to what extent lack of outdoor activity and excessive screen time are relevant to unhealthy weight gain among rural Chinese children and adolescents remain largely unknown.

The influence of lifestyle behaviors on childhood obesity is complex and may vary under different socio-demo-economic conditions. The rural-urban differences in the built environment, health and living resources and services challenge the over-extrapolation of associations between sedentary lifestyle and adiposity generated from high-income countries and urban areas to rural children and adolescents in low- and middle-income countries. Even though a number of studies have reported significant differences of physical activity and sedentary behaviors between rural and urban children and adolescents [15, 16], knowledge regarding the relationship between sedentary behaviors and childhood obesity in rural areas are limited. In order to guide effective and efficient preventions with limited resources, studies need to investigate how relevant and important of outdoor activity and screen time to obesity among rural children and adolescent in low- and middle-income countries.

In response to the urgent need for addressing the increasing threat of obesity among the young in less developed rural areas, this study focused on a population-based sample of seventh graders aged 12–15 years from a rural county in southwest China. The two primary aims were to 1) examine the prevalence of overweight/obesity, lack of outdoor activity (< 1 h per day) and excessive screen time (> 2 h per day) and to 2) investigate associations of lack of outdoor activity and excessive screen time with overweight/obesity. Generating findings directly from underrepresented rural adolescents would contribute to advocating more relevant and reliable public health efforts for tackling the obesity epidemic locally.

## Methods

### Sample and study design

Participants were from Mojiang, a rural county in Yunnan province, China. This study applied a cross-sectional design and used a population-based approach to invite all seventh graders and their parents from all 10 middle schools in Mojiang county using text messages and phone calls. A previous publication described the sampling in detail [17]. The present study excluded students aged younger than 12 or older than 15 to ensure age-representativeness of typical seventh graders, which resulted in 2264 participants (90.2% of the local population). All students and their adult caregivers provided assent and consent prior to participation. The research team conducted data collection at each school for two to three days in September and October 2014. The current study included anthropometric measurements conducted by trained research assistants and a questionnaire survey completed by participants with their parents. Research assistants distributed the take-home survey at school, instructed the adolescents to take the survey with their parents, and checked data quality of each returned survey. Adolescents or their parents were contacted to resolve missing responses and errors.

The study was conducted in accordance with the Declaration of Helsinki, and the protocol was approved by the Institutional Review Board of Kunming Medical University.

### Anthropometric measures

This study used body mass index (BMI) as an indicator of general obesity and waist circumferences as an indicator of central obesity. Research assistants received training to follow a standard protocol designed for this study. Weight was measured using a digital scale to the nearest 0.1 kg. Height was measured using a wall-mounted stadiometer to the nearest 0.1 cm. Waist circumference was measured using an inelastic measuring tape to the nearest 0.1 cm. BMI was calculated using weight divided by height squared (kg/m^2^). Overweight (≥ 85th percentile and < 95th percentile) and obesity (≥ 95th percentile) were defined using the age- and sex-specific BMI reference for Chinese school-aged children and adolescents developed by the Working Group on Obesity in China [18]. High waist circumference was defined as ≥90th percentile based on the age- and sex-specific reference of waist circumference for Chinese children and adolescents aged 7–18 years [19].

### Questionnaire survey

Adolescents reported time spent on outdoor activity and screen time using a take-home survey. The survey items were from the World Health Organization myopia risk factor questionnaire that had been applied among Singaporean adolescents [20]. Adolescents reported daily outdoor activity on weekdays and weekends separately. Daily outdoor activity on weekdays was assessed using “from Monday to Friday, the average time of your daily outdoor activity is: (1) less than one hour; (2) one hour or more; and (3) no outdoor activity.” The response options were recoded into 0.5, 1.5 and 0. Time of daily outdoor activity on weekends was assessed using “on weekends, the average time of your daily outdoor activity is: (1) less than one hour; (2) one to two hours; (3) two to three hours; (4) three hours or more; and (5) no outdoor activity”. The response options were recoded into 0.5, 1.5, 2.5, 3.5, and 0. Time of daily outdoor activity was calculated using weighted average of daily hours on weekdays and weekends. Based on the recommendation of having one hour or more daily physical activity, adolescents’ weekday, weekend and weighted average outdoor activity were categorized into < 1 h and ≥ 1 h, respectively.

Adolescents reported the average time that they spent on watching television, video games and computers on weekdays and weekends, respectively. The survey items were adapted from measures of children and adolescents’ screen-based behaviors in United States [21, 22], which have been translated and applied among Chinese adolescents [23]. The response options for each item were (1) less than half hour, (2) half hour to one hour, (3) one hour to two hours; (4) two to three hours; (5) more than three hours, and (6) none. These options were recoded into 0.3, 0.8, 1.3, 2.3, 3.3, and 0. Total screen time was summed from the three types of activities on weekdays and weekends, respectively. Daily screen time was calculated using weighted average of daily hours on weekdays and weekends. Based on the recommendation of limiting daily recreational screen time to ≤2 h, adolescents’ weekday, weekend, and weighted average screen time were categorized into ≤2 h and > 2 h, respectively.

The survey also contained questions asking adolescents to report frequencies of fruit intake and vegetable intake with the response options of everyday, often, sometimes, seldom and unknown and to write down the numbers of hours and minutes that they usually sleep in a day.

### Sociodemographic measurements

Adolescents reported their date of birth, sex, ethnicity, and number of siblings. Parents reported their education and occupation using the take-home survey. Adolescents’ age was calculated using date of birth and date of survey, and other sociodemographic items were recoded as binary variables.

### Statistical analysis

Statistical analyses were performed using SAS 9.4 (Cary, North Carolina, United States). Descriptive analyses (count, frequency, mean, standard deviation, etc.) were performed on all variables. Listwise deletion was used to address missing data in each analysis. T-tests were used to compare age differences by adolescents’ weight status (overweight/obese or not) and waist circumference (high or not). Chi-squares were used to examine differences in the prevalence of overweight/obesity and high waist circumferences by adolescents’ sex (boy vs. girl), being the single child (or not), ethnic minority (or not), parents’ highest education attainment (< high school vs. ≥ high school), fathers’ occupation (farmer vs. non-farmer), as well as adolescents’ outdoor activity (< 1 h vs. ≥ 1 h) and screen time (≤ 2 h vs. > 2 h). Furthermore, logistic regressions were performed to investigate odds ratios of overweight/obesity and high waist circumferences by adolescents’ outdoor activity and screen time, respectively, after adjusting for adolescents’ age, sex, being the single child, ethnic minority, frequencies of fruit intake and vegetable intake, sleep time (≥ 8 h per day or less), parents’ education, and fathers’ occupation. Then, logistic models kept both adolescents’ outdoor activity and screen time to examine joint effects on the prevalence of overweight/obesity and high waist circumferences, respectively.

## Results

Table 1 shows participants’ sociodemographic characteristics. This sample of rural seventh graders had a mean age of 13.7 years old. About half of them were boys, less than a quarter were the single child, and the majority were from ethnic minority groups. Most of their parents’ highest education attainment were below high school and about 60% of their fathers were farmers. The prevalence of overweight/obesity and high waist circumferences were 8.0 and 4.9%, respectively. Adolescents who were older and without siblings and whose parents had higher education attainment and whose fathers were not farmers were more likely to be overweight and obese. Adolescents who were girls and without siblings and whose parents had higher education attainment and whose fathers were not farmers were more likely to have high waist circumferences.

**Table 1:** Sociodemographic characteristics of the total sample and by weight status and waist circumference (*n* = 2264)

	All	Weight Status		p^a^	Waist Circumference		p^a^
		< 85th percentile	≥ 85th percentile		< 90th percentile	≥ 90th percentile	
Age	13.7 ± 0.7	13.7 ± 0.7	13.6 ± 0.7	.040	13.7 ± 0.7	13.7 ± 0.7	.715
Sex							
Boys	1164 (51.4%)	1074 (92.3%)	90 (7.7%)	.635	1121 (96.3%)	43 (3.7%)	.006
Girls	1100 (48.6%)	1009 (91.7%)	91 (8.3%)		1032 (93.8%)	68 (6.2%)	
Single child							
No	1741 (78.0%)	1626 (93.4%)	115 (6.6%)	<.001	1670 (95.9%)	71 (4.1%)	.002
Yes	490 (22.0%)	430 (87.8%)	60 (12.2%)		453 (92.5%)	37 (7.6%)	
Minority							
No	387 (17.1%)	354 (91.5%)	33 (8.5%)	.672	370 (95.6%)	17 (4.4%)	.610
Yes	1877 (82.9%)	1729 (92.1%)	148 (7.9%)		1783 (95.0%)	94 (5.0%)	
Parent education							
< high school	1678 (77.6%)	1567 (93.4%)	111 (6.6%)	<.001	1610 (96.0%)	68 (4.1%)	.001
≥ high school	485 (22.4%)	424 (87.4%)	61 (12.6%)		447 (92.2%)	38 (7.8%)	
Fathers’ work							
Not farmer	876 (39.3%)	778 (88.8%)	98 (11.2%)	<.001	818 (93.4%)	58 (6.6%)	.004
Farmer	1352 (60.7%)	1271 (94.0%)	81 (6.0%)		1299 (96.1%)	53 (3.9%)	

^a^ Comparisons were made using between-group t-tests and Chi-square tests

Table 2 demonstrates participants’ outdoor activity and screen time. Percentages of adolescents who had < 1 h daily outdoor activity were 43.7, 24.8 and 30.5% on weekdays, weekends, and weighted average days, respectively. Percentages of adolescents who had > 2 h daily screen time were 45.0, 68.9, and 52.4% on weekdays, weekends, and weighted average days, respectively. Prevalence of overweight/obesity was higher among adolescents who had < 1 h outdoor activity than those who had ≥1 h outdoor activity on weekdays (10.1% vs. 6.4%, *p* < .001) or weighted average days (10.9% vs. 6.8%, *p* = .001). Prevalence of high waist circumference was higher among adolescents who had < 1 h outdoor activity than those who had ≥1 h outdoor activity on weekdays (6.6% vs. 3.7%, *p* = .002) or weighted average days (6.8% vs. 4.1%, *p* = .006). Prevalence of high waist circumferences was also higher among adolescents who had > 2 h screen time than those had ≤2 h screen time on weekdays (6.0% vs. 4.1%, *p* =. 04) or weekends (5.7% vs. 3.2%, *p* = .01).

**Table 2:** Outdoor activity and screen time of the total sample and by weight status and waist circumference (n = 2264)

	All	Weight Status		p^a^	Waist Circumference		p^a^
		< 85th percentile	≥ 85th percentile		< 90th percentile	≥ 90th percentile	
Outdoor activity-weekday							
≥ 1 h	1260 (55.7%)	1180 (93.7%)	80 (6.4%)	.001	1214 (96.4%)	46 (3.7%)	.002
< 1 h	990 (43.7%)	890 (89.9%)	100 (10.1%)		925 (93.4%)	65 (6.6%)	
Outdoor activity-weekend							
≥ 1 h	1676 (74.0%)	1542 (92.0%)	134 (8.0%)	.886	1597 (95.3%)	79 (4.7%)	.446
< 1 h	562 (24.8%)	516 (91.8%)	46 (8.2%)		531 (94.5%)	31 (5.5%)	
Outdoor activity-weighted average							
≥ 1 h	1540 (68.0%)	1435 (93.2%)	105 (6.8%)	.001	1477 (95.9%)	63 (4.1%)	.006
< 1 h	691 (30.5%)	616 (89.2%)	75 (10.9%)		644 (93.2%)	47 (6.8%)	
Screen time-weekday							
≤ 2 h	1218 (53.8%)	1132 (92.9%)	86 (7.1%)	.089	1168 (95.9%)	50 (4.1%)	.041
> 2 h	1018 (45.0%)	926 (91.0%)	92 (9.0%)		957 (94.0%)	61 (6.0%)	
Screen time-weekend							
≤ 2 h	682 (30.1%)	636 (93.3%)	46 (6.7%)	.151	660 (96.8%)	22 (3.2%)	.013
> 2 h	1559 (68.9%)	1426 (91.5%)	133 (8.5%)		1470 (94.3%)	89 (5.7%)	
Screen time-weighted average							
≤ 2 h	1036 (45.8%)	963 (93.0%)	73 (7.1%)	.119	993 (95.9%)	43 (4.2%)	.088
> 2 h	1187 (52.4%)	1082 (91.2%)	105 (8.9%)		1119 (94.3%)	68 (5.7%)	

^a^ Comparisons were made using Chi-square tests

Table 3 shows odds ratios of adolescents being overweight/obese and having high waist circumferences by whether being lack of outdoor activity and having excessive screen time. Adolescents were more likely to be overweight and obese when they did not have ≥1 h outdoor activity on weekdays (OR: 1.86, 95% CI: 1.30, 2.66) and weighted average days (OR: 1.95, 95% CI: 1.36, 2.80). Similarly, adolescents were more likely to have high waist circumferences when they did not have ≥1 h outdoor activity on weekdays (OR: 2.22, 95% CI: 1.39, 3.57) and weighted average days (OR: 2.15, 95% CI: 1.36, 3.41). In terms of screen time, adolescents were more likely to have high waist circumferences when they had excessive screen time on weekends (OR: 2.08, 95% CI: 1.14, 3.80).

**Table 3:** Binary logistic regressions between adolescents’ weight status and waist circumferences and outdoor activity and screen time (n = 2264)

	Overweight/obesity		High waist circumference	
	OR	95% CI	OR	95% CI
Outdoor activity-weekday				
≥ 1 h	reference		reference	
< 1 h	1.86	(1.30, 2.66)**	2.22	(1.39, 3.57)**
Outdoor activity-weekend				
≥ 1 h	reference		reference	
< 1 h	1.31	(.87, 1.95)	1.45	(.88, 2.40)
Outdoor activity-daily average				
≥ 1 h	reference		reference	
< 1 h	1.95	(1.36, 2.80)**	2.15	(1.36, 3.41)**
Screen time-weekday				
≤ 2 h	reference		reference	
> 2 h	1.15	(.81, 1.65)	1.46	(.93, 2.32)
Screen time-weekend				
≤ 2 h	reference		reference	
> 2 h	0.96	(.64, 1.43)	2.08	(1.14, 3.80)*
Screen time-daily average				
≤ 2 h	reference		reference	
> 2 h	0.94	(.65, 1.36)	1.31	(.81, 2.11)

Covariates include adolescents’ age, sex, being the single child, ethnic minority, frequencies of fruit intake and vegetable intake, sleep time (≥ 8 h per day or not), parents’ education, and fathers’ occupation. * *p* < .05, ** *p* < .01

**Fig. 1:**
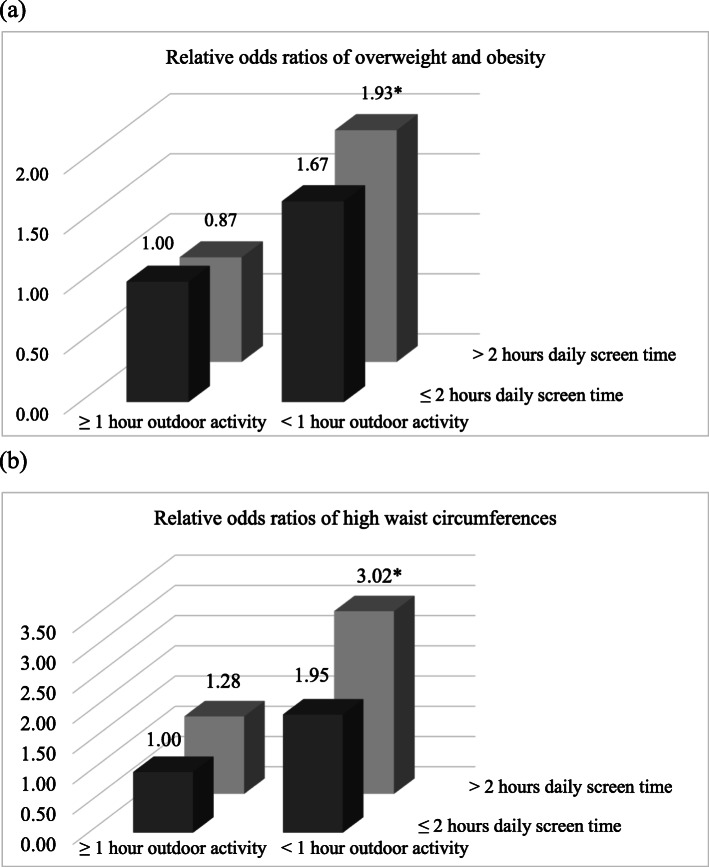
Joint associations of weighted daily physical activity and screen time with adolescents’ (**a**) weight status and (**b**) waist circumference, after adjusting for adolescents’ age, sex, being the single child, ethnic minority, frequencies of fruit intake and vegetable intake, sleep time (≥ 8 h per day or not), parents’ education, and fathers’ occupation (*p < 0.01)

Figure 1 shows the joint effects of weighted daily outdoor activity and screen time on adolescents’ weight status and waist circumference. Adolescents who did not meet both recommendations (≥ 1 h of weighted daily outdoor activity and ≤ 2 h of weighted daily screen time) had an increased risk of overweight/obesity than those met both recommendations (OR: 1.93. 95% CI: 1.15, 3.24). Similarly, adolescents who did not meet both recommendations had an increased risk of high waist circumferences than those met both recommendations (OR: 3.02, 95% CI: 1.54, 5.94).

## Discussion

Unlike previous research of childhood obesity that primarily conducted in economically developed countries and areas, this study exclusively focused on an underrepresented sample of early adolescents who were predominantly ethnic minorities living in rural southwest China. The study findings characterized the prevalence of overweight and obesity and high waist circumferences by key socio-demographic characteristics and demonstrated that not meeting the activity guidelines were associated with greater risks of childhood obesity.

Compared with rural school-aged children enrolled in the China Health and Nutrition Survey during a similar period, this sample had a substantially lower prevalence of overweight and obesity (19.2% vs. 8.0%) [4]. This finding may reflect the decreasing gradient of childhood obesity from northeast to southwest regions of China [24, 25]. It also indicated that the study participants may be from areas with a low prevalence of childhood obesity. Despite of this favoring obesity disparity, this sample demonstrated similar sociodemographic correlates of obesity that represented greater purchasing power as school-aged students living in urban China [26]. At the national level, panel analysis of 1985–2014 Chinese National Surveys on Students’ Constitution and Health found that increases in students’ weight were associated with per capital disposable income, and this positive association has increased persistently over the past 30 years [27]. At local level, findings from this study further indicated that adolescents from families with improved economic conditions may face greater risks in unhealthy weight gain.

Different from national data that showed greater obesity risk among boys than girls [4], this study found similar prevalence of overweight and obesity in both sexes. Moreover, the prevalence of high waist circumferences was higher in girls than in boys. This may attribute to sex differences in the growth patterns of body fat [28]. In general, childhood overweight and obesity in this sample of rural adolescents was characterized by a low prevalence with similar risks in boys and girls and higher risks in those from families with relatively higher socioeconomic status.

Outdoor time is positively associated with children’s physical activity and cardiorespiratory fitness [29]. In the present study, greater risks of overweight and obesity and high waist circumferences were consistently associated with having < 1 h of outdoor activities on weekdays and weighted average days, but not on weekends. The majority of these rural adolescents reported having adequate outdoor activity on weekends, which may lower the sensitivity to differentiate the potential effect of overall outdoor activity on adiposity. Nevertheless, outdoor activity on weekdays appeared to be an important indicator of overweight and obesity risk. A meta-analysis of four previous studies comparing meeting ≥1 h physical activity recommendation on weekdays and weekends found that Chinese students tended to be more active on weekdays than weekends [16]. This may because that the Chinese central government set national physical education guidelines for primary and sedentary schools [30], and students are more likely to have access to sport facilities during school days. Furthermore, the Youth Study of 2016 Physical Activity and Fitness in China showed that urban school students reported spending more time in moderate- to vigorous-physical activity than rural students [31]. Seventh graders in the present study were less active during school days than weekend, which may be due to the lack of adequate opportunities to be physically active during school days. This indicated a potential need to strengthen the implementation of national physical education guidelines and the promotion of outdoor activity in rural schools.

Studies investigating the impact of sedentary behaviors on pediatric adiposity often yielded inconsistent findings because the effect is hard to isolate from changes of other energy balance-related behaviors such as physical activity and dietary intake [32]. Similarly, this study only found that excessive screen time on weekends was associated with high waist circumferences. During weekdays, Chinese students generally spend most of their time on schoolwork due to the academic burden [33], which may weaken the obesogenic effect from the competing relationship between outdoor activity and screen time. However, when adolescents have more free time on weekend, their habitual choice of screen time over outdoor activity could contribute to long-term energy imbalance and weight gain. Regardless of the weekday-weekend discrepancies, this study demonstrated a consistent synergistic effect of lack of outdoor activity and excessive screen time on adolescents’ BMI status and waist circumferences. This finding suggests a potential need to promote active lifestyle for obesity prevention even among rural Chinese adolescents with a relatively low obesity prevalence.

The current study sample not only came from rural China but also predominantly consisted of ethnic minorities. Previous studies reported ethnicity variations in overweight and obesity among Chinese children and adolescents [34, 35]. However, factors associated with the ethnicity variations were not fully examined. Genetic susceptibility of excessive weight gain has been identified in Uyghur and Kazak Chinese living in Xinjiang, China [36]. Dietary cultures may contribute to the increased obesity risk in Mongolian and Manchu Chinese [37]. Even though the obesity risk among youth from ethnic groups of current study has been significantly lower than that of Han Chinese, their prevalence of overweight and obesity has been steadily increasing [35], which indicates the significant influence from modern lifestyles of the nutrition transition. As findings from the current study suggest, the favorable ethnicity variations in overweight and obesity among the current sample of minority youth may not compete with the influence of sedentary lifestyles.

The major limitation of this study was the cross-sectional design which does not support causality. Thus, this study cannot answer the question of whether a sedentary lifestyle contributes to adiposity or high BMI and waist circumferences make adolescents less active. This issue could be resolved by intervention studies examining the effect of promoting an active lifestyle by meeting the activity recommendations. In addition, the use of self-reported outdoor activity and screen time may introduce response bias due to memory errors and social desirability. Measurements using accelerometer and other wearable devices should be encouraged, although its administration at a large scale is a challenge. Combining objective and subjective measures by using accelerometer data from a subgroup of target population as calibrations could be a feasible alternative [38]. The confounding factors adjusted in the logistic models included socio-demographic factors, adolescents’ frequencies of fruit intake and vegetable intake, and sleep time. Other potential confounders, such as intake of energy-dense food, were not adjusted. The current study also assumed participants from regular schools to be general healthy and did not screen for health conditions that may influence adiposity. Moreover, cautions should be made when trying to generalize findings from this study to a different group of adolescents.

## Conclusions

Rural adolescents from southwest china had a relatively low prevalence of childhood obesity yet was subjected to similar sociodemographic disparities as adolescents living in urban or developed areas. Sedentary lifestyle was associated with overweight and obesity and high waist circumferences, which may attribute to the synergistic effect of lack of outdoor activity and excessive screen time. Findings from this study suggested potential strategies to promote healthy active lifestyle among rural adolescents. These include ensuring students have adequate outdoor activity during weekdays by strengthening the implementation of national physical education guidelines and preventing students having excessive screen time on weekends by engaging familial supports.

## Data Availability

Relevant data and material will be available from the corresponding author upon request.

## Abbreviations

BMI: Body mass index
OR: Odds ratio
CI: Confidence interval
